# Inhibition of Cariogenic Plaque Formation on Root Surface with Polydopamine-Induced-Polyethylene Glycol Coating

**DOI:** 10.3390/ma9060414

**Published:** 2016-05-25

**Authors:** May Lei Mei, Quan-Li Li, Chun Hung Chu

**Affiliations:** 1 Faculty of Dentistry, The University of Hong Kong, Hong Kong 999077, China; mei1123@hku.hk; 2 College of Stomatology, Anhui Medical University, Hefei 230032, China

**Keywords:** root caries, polydopamine, polyethylene, dentine, biofouling

## Abstract

Root caries prevention has been a challenge for clinicians due to its special anatomical location, which favors the accumulation of dental plaque. Researchers are looking for anti-biofouling material to inhibit bacterial growth on exposed root surfaces. This study aimed to develop polydopamine-induced-polyethylene glycol (PEG) and to study its anti-biofouling effect against a multi-species cariogenic biofilm on the root dentine surface. Hydroxyapatite disks and human dentine blocks were divided into four groups for experiments. They received polydopamine-induced-PEG, PEG, polydopamine, or water application. Contact angle, quartz crystal microbalance, and Fourier transform infrared spectroscopy were used to study the wetting property, surface affinity, and an infrared spectrum; the results indicated that PEG was induced by polydopamine onto a hydroxyapatite disk. Salivary mucin absorption on hydroxyapatite disks with polydopamine-induced-PEG was confirmed using spectrophotometry. The growth of a multi-species cariogenic biofilm on dentine blocks with polydopamine-induced-PEG was assessed and monitored by colony-forming units, confocal laser scanning microscopy, and scanning electron microscopy. The results showed that dentine with polydopamine-induced-PEG had fewer bacteria than other groups. In conclusion, a novel polydopamine-induced-PEG coating was developed. Its anti-biofouling effect inhibited salivary mucin absorption and cariogenic biofilm formation on dentine surface and thus may be used for the prevention of root dentine caries.

## 1. Introduction

Dental caries is the localized destruction of susceptible dental hard tissues by acidic by-products from the bacterial fermentation of dietary carbohydrates [1]. The disease process is initiated within the bacterial biofilm (dental plaque) that covers the tooth surface [2]. The development of dental plaque involves the adhesion of bacteria and subsequent colonization. The influence of the adsorbed proteins on bacteria adhesion has been suggested as playing a major role in bacteria–tooth interactions [3]. Compared to coronal caries (dental caries development in the tooth crown), the prevalence of root caries (dental caries development in the tooth root) is increasing. This can be due to extended root exposure time by increased life expectancy and the special anatomical location of the root in the oral cavity [4]. A systematic review concluded that about 40% of elderly people aged 70 suffered from untreated root caries [5]. Dentine on the root surface is soft and porous. Bacteria penetrate further into the tissue at an earlier stage of lesion development in root caries [6]. Thus, control of bacterial initial adhesion to the root surface is critical for the prevention of root caries [7,8].

Polyethylene glycol (PEG) was approved by the US Food and Drug Administration for internalization in the human body [9,10]. It is used as an anti-biofouling material to provide a hydrophilic environment on a substrate surface. Although the mechanism is not fully understood, it has been suggested that the hydrophilic surfaces could act as a good anti-biofouling barrier and that the protein and cell resistance of surface-immobilized PEG could be attributed to the large exclusion volume, high mobility, and steric hindrance effects of the highly hydrophilic layer [11,12]. PEG was shown to reduce protein adsorption and platelet adhesion in a blood-material interface [8]. A study synthesized a methacryloyloxydecyl phosphate PEG for the prevention of *Streptococcus mutans* adhesion on hydroxyapatite and found that its inhibitory effect on bacterial binding was diminished by saliva protein [13].

Mechanical plaque removal methods such as tooth brushing and dental flossing have been advocated as reducing the biofilm formation. However, these mechanical plaque removal methods require good manual dexterity and can be difficult to implement in certain circumstances, such as in older patients [2,14]. Exposed dentine on the root surface is more susceptible to caries than enamel. PEG can be used as an anti-biofouling material in inhibiting dental biofilm formation on the root dentine surface. Polydopamine is a bio-inspired polymer that can form a strong adhesive interaction with various substrates [15]. It also provides a versatile platform for secondary reactions for diverse functional applications [16]. Studies have indicated that polydopamine was effective in surface functionalization and biomolecule covalent immobilization [17,18,19,20]. The covalent immobilization of biomolecules maintained stable and long-term performance. The objectives of this study were to develop a polydopamine-induced-PEG coating method for the dentine surface and to investigate its anti-biofouling effect against a multi-species cariogenic biofilm.

## 2. Results

### 2.1. Characterization of the Polydopamine-Induced-PEG

**Figure 1:**
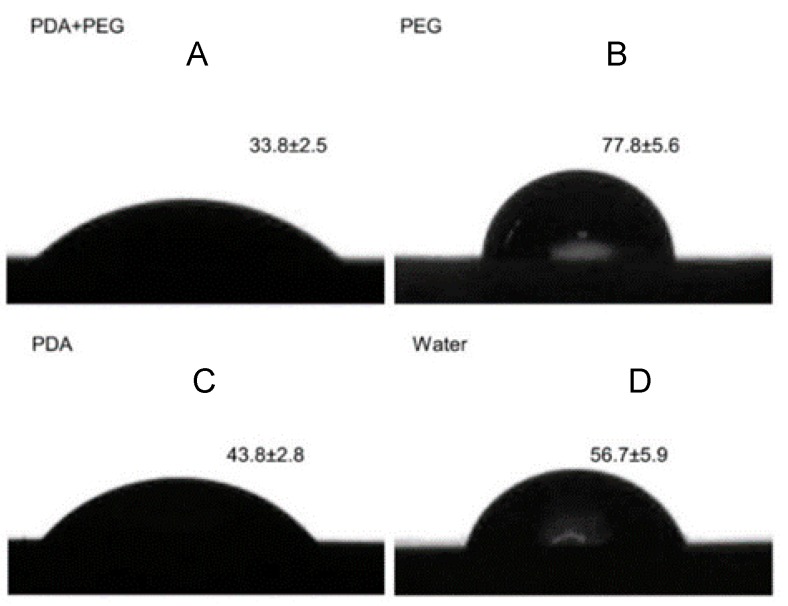
Contact angle (°) of PDA + PEG, PEG, PDA, and water on a hydroxyapatite disk. (**A**) PDA + PEG; (**B**) PEG; (**C**) PDA; (**D**) Water. (**A** < **C** < **D** < **B**; *p* < 0.001) PDA: polydopamine, PEG: polyethylene glycol.

The contact angles for Groups 1 to 4 were 33.8° ± 2.5°, 77.8° ± 5.6°, 43.8° ± 2.8°, and 56.7° ± 5.9°, respectively (*p* < 0.001; Figure 1). The multiple-comparison result is shown in Figure 1. The small contact angle of the hydroxyapatite disk treated with polydopamine and PEG indicated a strong hydrophilic property of the surface.

**Figure 2:**
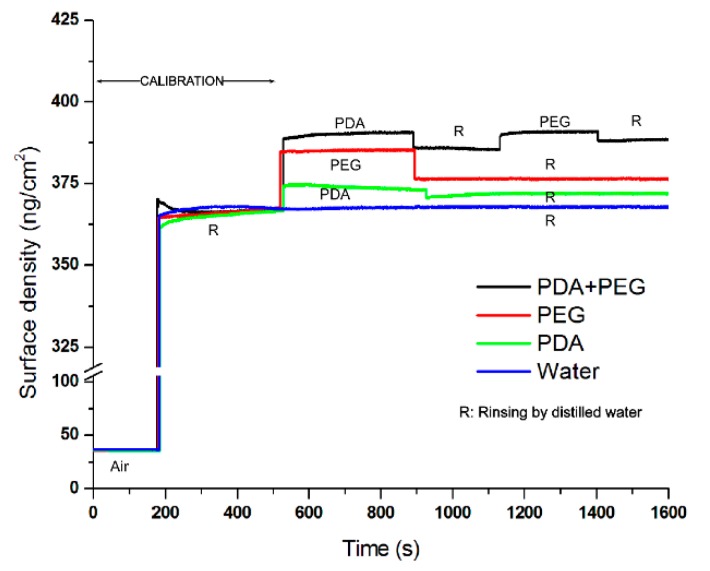
The surface density of quartz crystal with PDA + PEG, PEG, PDA, and water over time. PDA: polydopamine, PEG: polyethylene glycol.

**Figure 3:**
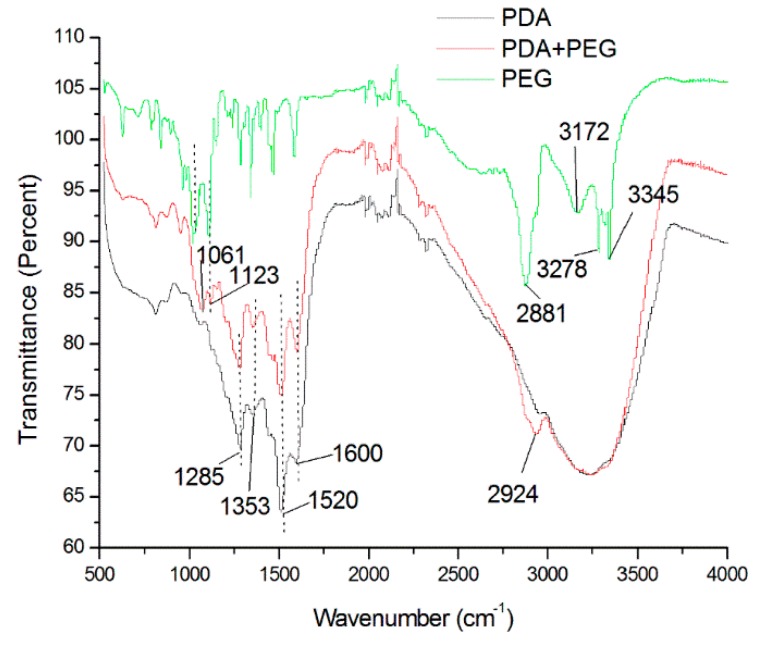
FTIR spectra of PDA + PEG, PDA, PEG treatments on a silicon disk. PDA: polydopamine, PEG: polyethylene glycol.

The change in the surface density of quartz crystal over time in Group 1 to Group 4 is shown in Figure 2. Results of the FTIR showed peaks at 1600 and 1353 cm^−1^ in the polydopamine spectrum, indicating aromatic ring chains and bonds between the phenyl groups in polydopamine, respectively (Figure 3, PDA group). For the PEG spectrum, the peaks at 1061 and 1123 cm^−1^ represented the C–O stretching vibration and the O–H bending vibration [21]. The peaks at 3172, 3278, and 3345 cm^−1^ represented free –OH and –NH_3_. These peaks were observed in the PEG spectrum but not in the dopamine + PEG spectrum. This suggested that no free –OH and –NH_2_ could be found in the dopamine + PEG treatment and that PEG was grafted chemically to polydopamine [22].

### 2.2. Mucin Absorption

The optical density from Groups 1 to 4 were 0.058 ± 0.007 ng/cm^2^, 0.115 ± 0.014 ng/cm^2^, 0.093 ± 0.01 ng/cm^2^, and 0.1 ± 0.015 ng/cm^2^, respectively (*p* < 0.01). The mucin absorbed by the hydroxyapatite disks of Group 1 was significantly less than that of Groups 2, 3, and 4.

### 2.3. Development of the Cariogenic Biofilm

**Figure 4:**
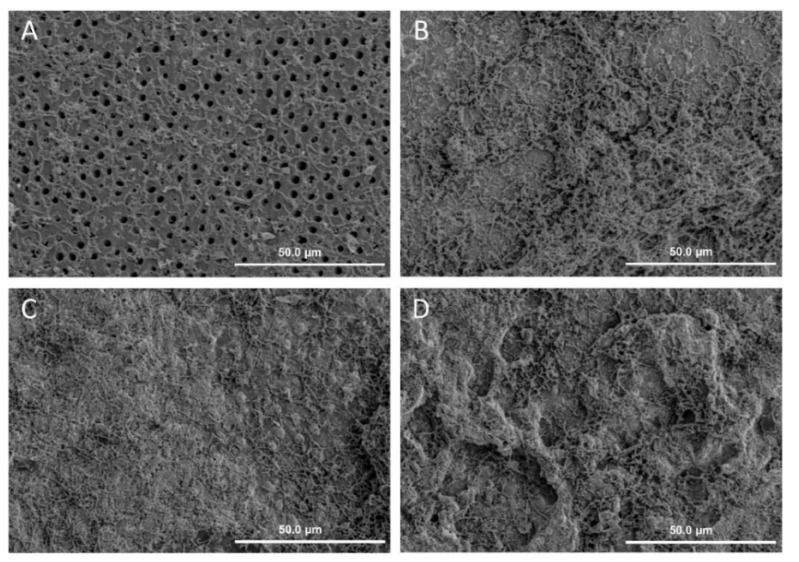
SEM images of biofilm of different groups on dentine surface after 48 h (×1000). (**A**) PDA + PEG; (**B**) PEG; (**C**) PDA; (**D**) Water (PDA: polydopamine, PEG: polyethylene glycol).

The surface morphology of the cariogenic biofilm on the dentine surface after 48 h under SEM is shown in Figure 4. A monolayer of biofilm was found in Group 1. The bacteria were sparsely distributed on the dentine surface. The openings of dentinal tubules were visible. A dense biofilm with confluent bacteria covering the dentine surface was observed in Groups 2 to 4.

**Figure 5:**
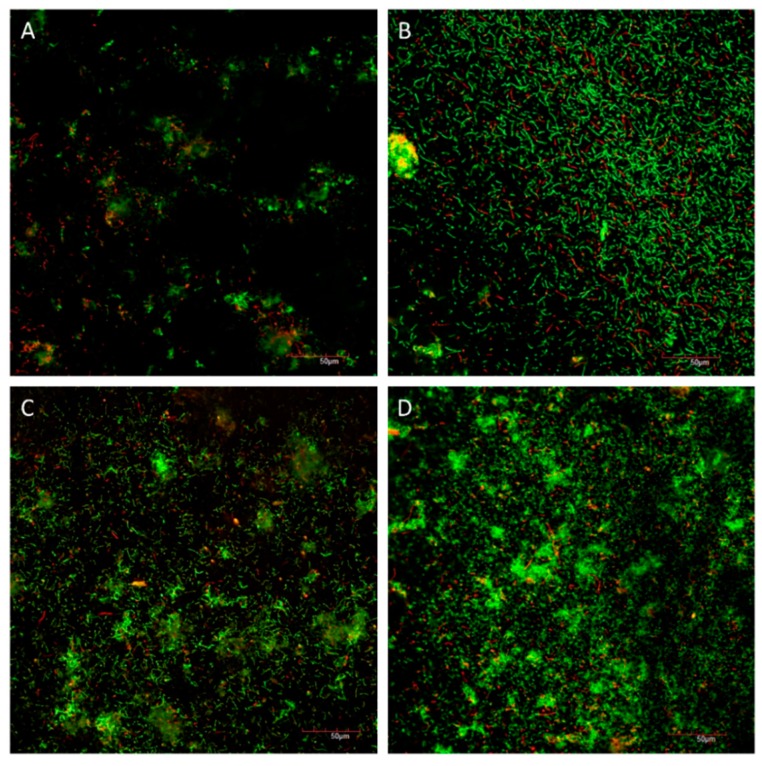
CLSM images of biofilm of different groups on dentine surface after 48 h (×400). (**A**) PDA + PEG; (**B**) PEG; (**C**) PDA; (**D**) Water (PDA: polydopamine, PEG: polyethylene glycol).

Figure 5 showed CLSM images of the live/dead staining of different groups on the dentine surface after 48 h. The red-to-green ratio of Groups 1 to 4 were 0.12 ± 0.05, 0.19 ± 0.05, 0.11 ± 0.04 and 0.04 ± 0.02, respectively (*p* = 0.001). Multiple comparisons showed that the red-to-green ratio of Group 4 was smaller than that of the other groups, and the largest red-to-green ratio was shown in Group 2.

The log (CFU/mL) of Groups 1 to 4 were 4.9 ± 0.4, 5.8 ± 0.1, 5.7 ± 0.1, and 5.8 ± 0.2, respectively (*p* < 0.05). Multiple comparisons showed the log (CFU/mL) of Group 1 was smaller than that of the other groups, and there was no significant difference in log (CFU/mL) among Groups 2, 3, and 4.

## 3. Discussion

The results of the experiments on contact angle and QCM suggested that a novel polydopamine-induced-PEG coating on the dentine surface was developed in this study. This polydopamine-induced-PEG coating had the anti-biofouling effect in inhibiting salivary mucin absorption and cariogenic biofilm formation. The substrates used in this study are hydroxyapatite disks and human dentine blocks. The hydroxyapatite disks were standardized and made from hydroxyapatite powder. They were used for the assessment of the contact angle and mucin absorption because these two assessments require high test–retest variability. Autoclaving was used to sterilize both hydroxyapatite disks and dentine blocks because this would render the teeth free of viable microorganisms [23]. Although autoclaving of teeth may reduce dentine microhardness, an *in vitro* study has shown that it did not significantly alter the physical properties [24]. Contact angle quantifies the wettability of the polydopamine-induced-PEG-coated hydroxyapatite by water via the Young equation. Spectrophotometry is widely used for the study of chemical substances and can determine the amount of mucin absorbed on the hydroxyapatite disk through calculations of observed wavelengths. The early stage of bacterial invasion in the caries process involves *Streptococci*, *Lactobacilli*, and *Actinomycetes*. *Streptococcus mutans* is the most important odontopathogens involved in the initiation and progression of caries. *Actinomyces israelii* has the potential to invade dentinal tubules and is associated with root surface caries. *Lactobacillus acidophilus* is one of the most abundant species frequently found in both superficial deep carious lesions [25]. For the foregoing reasons, these three cariogenic species were chosen to form a multi-species cariogenic biofilm. It is noteworthy that dental caries is a polymicrobial infection process caused by over 700 species of oral bacteria [26]. In addition, this study incubated the bacteria on the root dentine surface with no prior saliva contamination. The results of this *in vitro* study need to be interpreted with caution.

After we confirmed that PEG connected to hydroxyapatite via dopamine, human dentine blocks were used to study the inhibition effect of this polydopamine-induced-PEG coating on dentine against a cariogenic biofilm formation. The CFU and SEM results corroborated that dopamine-induced-PEG had inhibited the growth of a cariogenic biofilm on the dentine surface. Rinsing with distilled water did not notably reduce the surface density after the polydopamine-induced-PEG treatment. This suggested covalent binding between the polydopamine and PEG, which made the coating surface more stable.

QCM is a well-suited technique for monitoring mass attached to coating equipment in a vacuum. It can be used for the investigation of the adsorption and surface reaction in the monolayer range via changes in the resonant frequency [27,28]. The substrate of QCM is standard quartz crystal, and this is used to detect minute changes on the surface at the nano-gram level. Since no hydroxyapatite substrate was used, the results of QCM could only indirectly indicate the binding between polydopamine and PEG based on changes in the surface density. A FTIR study is necessary to determine whether PEG reacted with polydopamine. In addition, the results of the contact angle assessment demonstrated that the polydopamine-induced-PEG modified the hydroxyapatite surface by making it more hydrophilic.

**Figure 6:**
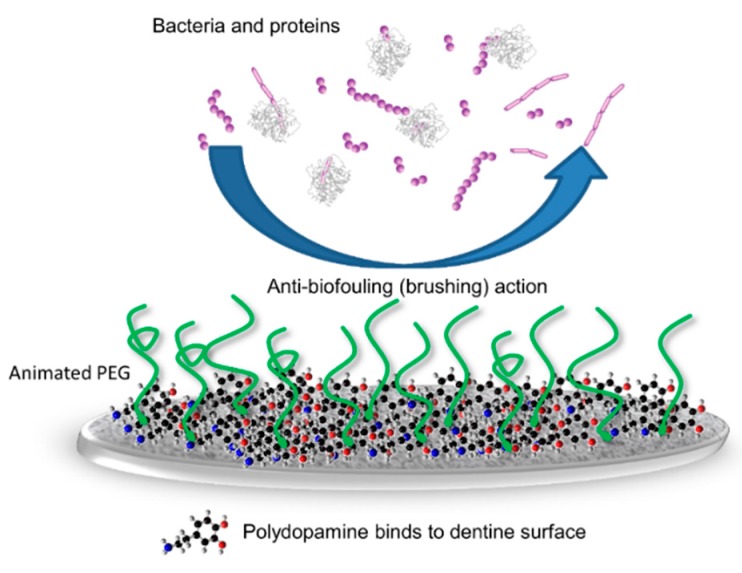
Schematic diagram illustrating the anti-biofouling of polydopamine-induced-polyethylene glycol (PEG).

PEG is a water-swellable, non-toxic, and biocompatible polymer. Its use in biotechnology has been reported in the literature [8,11,29]. Polymer brushes consisting of PEG opened a wide door in biomaterials research due to the suitable properties of the polymer [30]. Polymer brushes are linear polymer chains terminally anchored to solid surfaces. If the distance between the anchoring points of the surface-grafted chains is small, interchain correlations occur. The tethered chains are stretched away from the surface leading to a “brush”-like conformation. Such polymeric monolayers play an important role in a wide range of colloid stabilization, tribology, lubrication, and rheology [30]. PEG-modified surfaces have a high degree of hydration. The modified surfaces are effective in reducing diatom adhesion and weakening protein adsorption [12,31]. This study showed that the tooth surface became more hydrophilic after coating it with polydopamine-induced-PEG. The resistance to adhesion of protein and bacteria to the PEG-modified surface can be attributed to the large exclusion volume, high mobility, and steric hindrance effects of this hydrophilic layer (Figure 6) [12,32].

**Figure 7:**
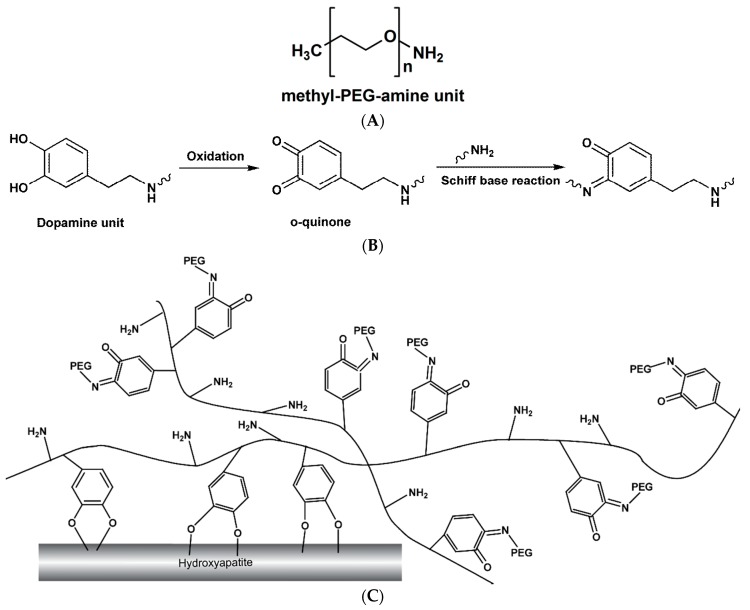
Schematic diagram illustrating interfacial location of aminated polyethylene glycol, polydopamine, and hydroxyapatite. (**A**) methyl-polyethylene glycol (PEG)-amine unit; (**B**) Immobilization of selenocystamine via polydopamine linker [38]; (**C**) Interfacial location of aminated PEG, polydopamine and hydroxyapatite [15].

The bonding of adhesives to enamel is reliable and strong because enamel is highly mineralized. However, the bonding of adhesives to dentine is less satisfactory because dentine contains, by weight, 20% organic material (mainly collagens) and 10% water. Mussels can attach to various surfaces in aqueous conditions, ranging from natural inorganic materials and organic materials to synthetic materials [16]. Such adhesive properties rely on exhaustively repeated 3,4-dihydroxy-L-phenylalanine (DOPA) and amine [33,34]. Dopamine was identified as a small-molecule compound that contains both DOPA and amine (Figure 7). It has strong and good biodegradability and low cytotoxicity [35]. The oxidative polymerization of dopamine in aqueous solutions spontaneously forms polydopamine [36]. It mimics repeated DOPA and amine and therefore exhibits a strong adhesive property in relation to various substrates under wet conditions [36,37]. Polydopamine is used for biomaterial surface modification because it is easy to obtain abundant active groups. These active groups are mostly phenolic hydroxyl/o-quinone and amino/imino for bimolecular immobilization on the material surface with minimal change in the chemical structure of biomaterials [38]. The plausible mechanism of polydopamine-induced-PEG coating is shown in Figure 7. Polydopamine connects PEG with an active group of o-quinone. It can also attach to hydroxyapatite with phenolic hydroxyl (Figure 7c). This mechanism could be the reason for the promising anti-biofouling effect of polydopamine-induced-PEG coating on dentine.

Mucins are the fundamental organic components of mucus in human saliva. It remains controversial whether mucin reduces or enhances cariogenic bacterial adhesion [39]. However, mucin may act as a receptor for accumulation and biofilm formation, and it plays an important role in the agglutination/aggregation of a number of microorganisms [1,40,41]. The mucin absorption measured by a spectrophotometer in this study demonstrated that polydopamine-induced-PEG coating inhibited mucin adhesion to hydroxyapatite. This can be one of the main reasons for the biofilm-inhibitory effect of polydopamine-induced-PEG. Likewise, polydopamine-induced-PEG showed an inhibitory effect on cariogenic biofilm formation on dentine. The total bacterial amount of the polydopamine-induced-PEG group was significantly lower than that of the other three groups, and the quantity of the biofilm does increase with time. The reason might be that the “micro-brush” effect of polydopamine-induced-PEG coating is a mechanical effect rather than a chemically antimicrobial function. It is plausible that the accumulation of bacteria on the dentine surface weakened the “micro-brush” effect by occupying the space for PEG movement, which in turn caused more bacteria to settle on the dentine surface. Thus, it is difficult to rely completely on this function to protect the tooth surface due to the complex environment of oral biofilm. The polydopamine-induced-PEG should be applied more frequently to sustain the anti-biofouling effect. Polydopamine-induced-PEG may be used as an anti-biofouling agent in mouth rinse and to prevent cariogenic biofilm formation after teeth cleaning. Another clinical application involves adding it to a varnish to protect the tooth from biofilm adhesion. This *in vitro* study demonstrated the promising results of a novel anti-biofouling method to help control cariogenic biofilm formation. Further studies aiming to simplify the coating process and to sustain the coating are needed.

## 4. Materials and Methods

### 4.1. Dentine Block and Hydroxyapatite Disk Preparation

This study was approved by the Institutional Review Board of the University of Hong Kong/Hospital Authority Hong Kong West Cluster (IRB UW13-555). Extracted sound human third molars were collected with patient consent. They were stored in distilled water at 4 °C and were used within one month of extraction. Thirty dentine blocks of 2 × 2 × 4 mm^3^ were prepared from a tooth root modified from our previous protocol [42]. The blocks were treated with 1% acetic acid for 5 s and ultrasonically washed with deionized water to remove the smear layer [43]. In addition, 36 hydroxyapatite disks were prepared by compressing hydroxyapatite powder (Sigma-Aldrich Co., St. Louis, MO, USA) into a circular mold (12 mm in diameter, 1 mm in thickness). They were sintered at 900 °C for 2 h. The surfaces of the hydroxyapatite disks were polished using micro-fine 4000 grid sanding paper. Commercially available methyl-PEG-amine (mPEG-NH_2_ Mw = 2000) (Shanghai Science Peptide Biological Technology Co., Ltd., Shanghai, China) and polydopamine (Sigma-Aldrich, St. Louis, MO, USA) were used in this study.

### 4.2. Experimental Design

The dentine blocks (2 × 2 × 4 mm^3^) and hydroxyapatite disks (diameter: 10 mm) were autoclaved for sterilization. They were randomly and equally divided into 4 experimental groups. In Group 1, dentine blocks and hydroxyapatite disks were immersed in 2 mg/mL of freshly prepared polydopamine solution (in 10 mM Tris buffer, pH = 8.5) at 23 °C for 12 h in the dark [16]. After rinsing for 10 min with Tris buffer to remove non-attached polydopamine and drying under nitrogen, the blocks and the disks were then immersed in a freshly prepared solution of 1 mg/mL of mPEG-NH_2_ (in 10 mM Tris buffer, pH = 8.5) at 23 °C for 4 h. They were then rinsed with Tris buffer for another 10 min to remove any extra solution [16]. In Group 2, dentine blocks and hydroxyapatite disks were immersed in a freshly prepared solution of 1 mg/mL of mPEG-NH_2_ at 23 °C temperature for 4 h. They were rinsed with Tris buffer for 10 min to remove residual solution. In Group 3, dentine blocks and hydroxyapatite disks were immersed in 2 mg/mL of freshly prepared polydopamine solution at 23 °C for 12 h in the dark. They were rinsed with Tris buffer for 10 min to remove nonattached polydopamine. In Group 4, dentine blocks and hydroxyapatite disks were immersed in distilled water.

### 4.3. Characterization of the Polydopamine-Induced-PEG

#### 4.3.1. Contact Angle Measurement

The degree of wetting (wettability) of the hydroxyapatite disks with coating was evaluated by measuring the contact angle using a drop-shape analyzer (DSA100. Krüss GmbH, Hamburg, Germany) equipped with a pendant drop module. It quantifies the wettability of the hydroxyapatite samples by water via the Young equation. Three replicates were measured for each group, and three samples per group were assessed. Each water drop (5 µL) was deposited onto the disk surface and kept for 15 s. Then, an image of the drop was taken by a built-in camera and analyzed using the software (Image J 1.51a, National Institutes of Health, Bethesda, MD, USA) supplied by the manufacturer. All measurements were conducted at room temperature.

#### 4.3.2. Quartz Crystal Microbalance

The affinity of the polydopamine-induced-PEG to the surface was studied by quartz crystal microbalance (QCM; Seiko QCM 922, Princeton Applied Research Inc., Oak Ridge, TN, USA). Mass variation before and after coating was measured. The change of the effective surface mass on the quartz crystal altered the gold resonant frequency. The differences in the resonant frequency were recorded as a function of the potential, and the corresponding mass changes were calculated. The experimentally determined Sauerbrey constant for the gold resonator was 176.0 Hz cm^2^·µg^−1^ [44].

QCM study was carried out with 0.198-cm^2^ standard-finished 9-MHz AT cut gold resonators (Princeton Applied Research Inc., Oak Ridge, TN, USA) sputtered on quartz. All the experiments were conducted by dipping the quartz crystal into 4 mL of experimental solutions in a 16-well plate at 23 °C. The quartz crystal was dipped into a sequence of reagents, which is described in Section 2.2 for Groups 1–4. Distilled water (pH = 7.0) was used to remove the unstable reagent from the quartz crystal surface. The real-time frequency changes during the experiment were recorded using WinQCM computer software (Princeton Applied Research Inc., Oak Ridge, TN, USA). The surface density of the quartz crystal was calculated.

#### 4.3.3. Fourier Transform Infrared Measurement

The infrared spectrum of the absorption of the standard silicon disk with different treatments were assessed by means of a Fourier transform infrared (FTIR) spectrometer (Nicolet 8700, Thermo Scientific Instrument Co., Hudson, NH, USA).

### 4.4. Absorption of Mucin

Absorption of mucin on the surface of the hydroxyapatite disks with coating was evaluated. The disks were immersed in 10 mg/mL of mucin obtained from bovine submaxillary glands (Sigma–Aldrich, Co., St. Louis, MO, USA) for 90 min. They were rinsed with phosphate buffer solution (PBS) to remove the residual mucin before they were stained with Alcian blue solution (pH = 2.5). The absorbed mucin was then extracted using 30% hydrogen peroxide. The optical density of the supernatant (200 mL) was measured spectrophotometrically at 595 nm [45]. The expected difference is at least 10°. Six hydroxyapatite disks per group were studied.

### 4.5. Development of the Cariogenic Biofilm

A multi-species cariogenic biofilm was developed using a method modified from our previous study [25]. Briefly, *Streptococcus mutans* from the American Type Culture Collection (ATCC) 35668, *Lactobacillus acidophilus* ATCC 9224, and *Actinomyces israelii* were anaerobically cultured on blood agar plates at 37 °C for 48 h. A single colony was picked from each plate to prepare 24-h broth cultures in brain heart infusion (BHI) supplemented with 5% sucrose at 37 °C under anaerobic conditions (95% nitrogen and 5% carbon dioxide). Subsequently, bacterial cell pellets were harvested and resuspended in the BHI to a cell density of McFarland 2 (106 cells/mL). A 500-µL aliquot of each bacteria culture was mixed and inoculated on each dentine block sitting in a well of a 24-well plate with BHI. The dentine blocks were aerobically incubated in 1 mL of mixed BHI suspension (adhesion phase) at 37 °C for 90 min. After rinsing with PBS to remove non-adherent cells, the dentine blocks were aerobically incubated in the BHI at 37 °C (biofilm phase). The dentine blocks were taken out after 48 h for analysis; the BHI was refreshed after 24 h [45].

#### 4.5.1. Morphology of the Cariogenic Biofilm

Scanning electron microscopy (SEM) was used to monitor the topographical features of the biofilm. In preparation for SEM, the dentine blocks with biofilm were rinsed in 4% formaldehyde followed by 1% PBS; they were then placed in 1% osmium tetroxide solution for 60 min. The blocks were washed in distilled water and dehydrated in a series of ethanol solutions at increasing concentrations (70% for 10 min, 95% for 10 min, and 100% for 20 min). The blocks were dried in a desiccator and sputter-coated with gold. The surface topographies of biofilms were studied under SEM (Hitachi S-4800 FEG Scanning Electron Microscope, Hitachi Ltd., Tokyo, Japan) at 12 kV in high-vacuum mode [25]. Two dentine blocks per group were used in this qualitative experiment.

#### 4.5.2. Viability of the Cariogenic Biofilm

Confocal laser scanning microscopy (CLSM) (Fluoview FV 1000, Olympus, Tokyo, Japan) was employed to assess the viability of the biofilms. The bacteria on the dentine surfaces were labeled *in situ* using two fluorescent probes: propodium iodide and SYTO-9 dye (LIVE/DEAD BacLight Bacterial viability kit, Molecular Probes, Eugene, OR, USA). Five images from the middle layer of each biofilm were obtained using CLSM and were analyzed using Image J (Version 1.51a, National Institutes of Health, Bethesda, MD, USA). The red-to-green ratio was calculated to denote the ratio of dead-to-live bacteria, respectively [25]. Two dentine blocks per group were used in this qualitative experiment.

#### 4.5.3. Kinetics of the Cariogenic B**iofilm**

Serial 10-fold dilutions of homogenized biofilm samples in 1% PBS were collected from dentine blocks and then plated in duplicate with a spiral plater (Autoplate 4000; Spiral Biotech Inc., Norwood, MA, USA) onto horse blood agar (Defib Horse Blood; Hemostat Laboratories, Dixon, CA, USA). The plates were incubated aerobically for 72 h to assess the bacterial colony-forming unit (CFU) per mL. Biofilms on six dentine blocks per group were studied.

### 4.6. Statistical Analysis

A Shapiro–Wilk test was used to assess the data for normality. One-way ANOVA was performed to compare the contact angle, mucin absorption, and log CFU among the four experimental groups. A Bonferroni test was carried out for multiple comparisons of the result. All the analyses were conducted using IBM SPSS version 2.0 software (IBM Corporation, Armonk, New York, NY, USA). The cut-off level for significance was taken as 5% for all the analyses.

## 5. Conclusions

A novel polydopamine-induced-PEG coating on hydroxyapatite was developed. This coating has a dental anti-fouling effect in terms of the inhibition of salivary mucin absorption and cariogenic biofilm formation.

## Abbreviations

The following abbreviations are used in this manuscript:

PEG	polyethylene glycol
QCM	quartz crystal microbalance
ATCC	American Type Culture Collection
PBS	phosphate buffer solution
BHI	brain heart infusion
SEM	scanning electron microscopy
CFU	colony-forming unit
ANOVA	analysis of variance
DOPA	3,4-dihydroxy-L-phenylalanine
